# Random Error Analysis of MEMS Gyroscope Based on an Improved DAVAR Algorithm

**DOI:** 10.3390/mi9080373

**Published:** 2018-07-27

**Authors:** Jinlong Song, Zhiyong Shi, Lvhua Wang, Hailiang Wang

**Affiliations:** Department of Vehicle and Electrical Engineering, Army Engineering University, Shijiazhuang 050003, China; sjzsong_jl@163.com (J.S.); 18222885931@163.com (L.W.); m18731697828@163.com (H.W.)

**Keywords:** dynamic Allan variance, kurtosis, sliding kurtosis contribution coefficient, MEMS

## Abstract

In view that traditional dynamic Allan variance (DAVAR) method is difficult to make a good balance between dynamic tracking capabilities and the confidence of the estimation. And the reason is the use of a rectangular window with the fixed window length to intercept the original signal. So an improved dynamic Allan variance method was proposed. Compared with the traditional Allan variance method, this method can adjust the window length of the rectangular window adaptively. The data in the beginning and terminal interval was extended with the inverted mirror extension method to improve the utilization rate of the interval data. And the sliding kurtosis contribution coefficient and kurtosis were introduced to adjust the length of the rectangular window by sensing the content of shock signal in terminal interval. The method analyzed the window length change factor in different stable conditions and adjusted the rectangular window’s window length according to the kurtosis, sliding kurtosis contribution coefficient. The test results show that the more the kurtosis stability threshold was close to 3, the stronger the dynamic tracking ability of DAVAR would be. But the kurtosis stability threshold was too close to 3, there was a misjudgement in kurtosis analysis of signal stability, resulting in distortion of DAVAR analysis results. When using the improved DAVAR method, the kurtosis stability threshold can be close to 3 to improve the tracking ability and the estimation confidence in stable interval. Therefore, it solved the problem that the dynamic Allan variance tracking ability and confidence level were difficult to take into account, and also solved the problem of misjudgement in the stability analysis of kurtosis.

## 1. Introduction

Micro electromechanical system (MEMS) gyroscope has the advantages of small size, light weight, and low cost [1,2,3,4], which is widely used in various fields of modern navigation. However, the accuracy of MEMS gyroscopes is low. So it is important to compensate the errors of MEMS gyroscopes.

MEMS gyroscope error can be divided into deterministic error and random error [5]. Deterministic error can be eliminated by calibrating the MEMS gyroscope [6]. The random error has a strong randomness, so the random error becomes one of the main factors that limit the accuracy of the MEMS gyroscope. At present, the modeling methods of random error about MEMS gyroscopes mainly include autocorrelation analysis, power spectral density method and Allan variance analysis method. Allan variance analysis can characterize and identify various random error sources of MEMS gyroscopes. It is convenient to establish accurate random error model and improve navigation precision. Many scholars such as Gao et al., Chen et al. and Li et al. have made Allan variance, overlapped Allan variance identification for random errors of MEMS gyroscopes and achieved good results [7,8,9]. However, MEMS gyroscope signals are non-stationary, so the Allan variance is difficult to track the dynamic changes of the signal. Wang et al. use a rectangular window to intercept the original signal and analyze the Allan variance for intercepted signal. So the time-varying characteristics of various random errors can be effectively tracked [10]. However, the DAVAR method intercepts the original signal using a fixed length rectangular window which has a defect of power leakage and low confidence in identifying noise. In Zhang et al. paper [11], the original signal is separated according to the high and low frequency by using the wavelet decomposition method. And next, the signals with different frequencies were used different window functions to intercept the original signal, which reduce the power loss of DAVAR method [11]. Zhu et al. adjust the length of the rectangular window according to the original signal kurtosis and the changes of variance to improve the confidence level of the variance estimation effectively [12,13]. But the kurtosis and variance reflect the stability of the rectangular window interception signal, so when the length of the window is changed according to the variance and kurtosis, there are defects in the lag of window length regulation. In Gu et al. paper [14], the choice of the window length is based on a fuzzy control method, which improves the dynamic tracking ability of DAVAR. In Zhu et al. paper [15], the effective length of the analysis data is increased with inverted mirror extension method, however, all signals are extended and analyzed, which is difficult to ensure that the analysis results reflect the true characteristics of the original signal.

Because the dynamic Allan variance analysis which use the rectangular window with a fixed window length to intercept the original signal has the disadvantage of power leakage and insensitive tracking of the impact components in the signal, so the paper proposes a method which uses sliding kurtosis contribution coefficient to sense the impact component at the end of the intercepted signal, and use the sliding kurtosis contribution coefficient and kurtosis together to control the rectangular window length. And the extension enhances the beginning and ending signal utilization with mirror inverted. Through the simulation, it is found that the sliding kurtosis contribution coefficient can sense the content of the impact component at the end of the intercepted signal so that the window length of the rectangular window can be adjusted in time, which enhances the sensitivity of the dynamic Allan variance analysis to the impact signal. Finally, the feasibility of the proposed method is verified through the actual measurement and the analysis of MEMS gyroscope signals.

## 2. Allan Variance Analysis and Dynamic Allan Variance Analysis

### 2.1. Allan Variance Analysis

Allan variance analysis is a method of analyzing the frequency of signal stability in the time domain, which was originally proposed by David Allan of the National Bureau of Standards (NBS) [16,17]. The Allan variance is the most often used method for reducing a clock-noise time series to a statistical summary of frequency stability, its use has also spread to other fields of science as a tool for studying low-frequency spectral behavior of physical processes [18]. Random drift of MEMS gyroscope has similar statistical characteristics. Therefore, many scholars also use the Allan variance analysis to identify the random error of the MEMS gyroscope. It is defined that the sampling time $\tau_{0}$ of MEMS gyroscope to obtain *N* sampling points, the sampling sequence {ω} is divided into *K* groups, *K = N/M*. Each group contains *M* data and the relevant time $\tau =M\tau_{0}$ is the total sampling time of each group.

The mean of each group is:

$${\bar{\omega }}_{k}(M)=\frac{1}{M}\sum_{i=1}^{M}\omega_{(k-1)M+i},(k=1,2,\cdots K)\;$$

Allan variance is defined as shown in Equation (1).

(1) $$\sigma_{A}^{2}(\tau )\cdot \frac{1}{2}\langle {\left({\bar{\omega }}_{k+1}(M)-{\bar{\omega }}_{k}(M)\right)}^{2}\rangle =\frac{1}{2(K-1)}\sum_{k=1}^{K-1}{\left({\bar{\omega }}_{k+1}(M)-{\bar{\omega }}_{k}(M)\right)}^{2}\;$$

In Equation (1), <> represents the overall average of the data.

MEMS gyroscope random error usually contains angle random walk (ARW, A is a coefficient), bias instability (BI, B is a coefficient), rate random walk (RRW, R is a coefficient), rate ramp (RR, K is a coefficient), quantization noise (QN, Q is a coefficient), Markov noise (MN) and sinusoidal noise (SN). Due to the different types of gyroscopes and test environments, random noise of various components may exist in the output data. It is hypothesized that the noise is independent of each other and the Allan variance can be expressed as the sum of squares of the random errors, as follows:

$$\sigma^{2}(\tau )=\sigma_{ARW}^{2}(\tau )+\sigma_{BI}^{2}(\tau )+\sigma_{RRW}^{2}(\tau )+\sigma_{QN}^{2}(\tau )+\sigma_{RR}^{2}(\tau )+\sigma_{SN}^{2}(\tau )+\sigma_{MN}^{2}(\tau )+\cdots \;$$

Among them, the Allan variance of the five random errors including QN, ARW, BI, RRW, and RR is as following Equations (2)~(6). Each error source can be expressed as proportional to the −2~2 power of the form, therefore, Allan variance can be expressed as Equation (7).

(2) $$\sigma_{QN}^{2}(\tau )=\frac{3Q^{2}}{\tau^{2}}\;$$

(3) $$\sigma_{ARW}^{2}(\tau )=\frac{N^{2}}{\tau }\;$$

(4) $$\sigma_{BI}^{2}(\tau )=\frac{B^{2}\cdot 2\ln2}{\pi }\;$$

(5) $$\sigma_{RRW}^{2}(\tau )=\frac{P^{2}\tau }{3}\;$$

(6) $$\sigma_{RR}^{2}(\tau )=\frac{R^{2}\tau^{2}}{2}\;$$

(7) $$\sigma^{2}(\tau )=\sum_{i=-2}^{2}A_{i}\tau^{i},\;(i=-2,-1,0,1,2)\;$$

**Figure 1:**
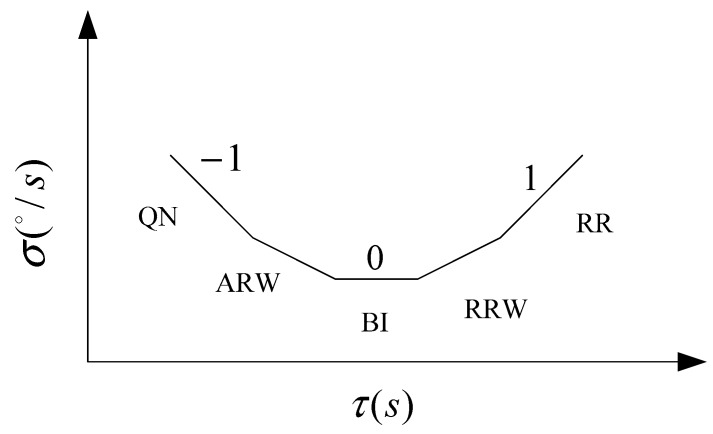
Allan standard deviation curve of MEMS gyroscope signal.

Five random errors in the σ(τ)−τ logarithmic curve are shown in Figure 1. σ(τ) is Allan standard deviation.

### 2.2. Allan Variance of Analog MEMS Gyroscope Signal

**Figure 2:**
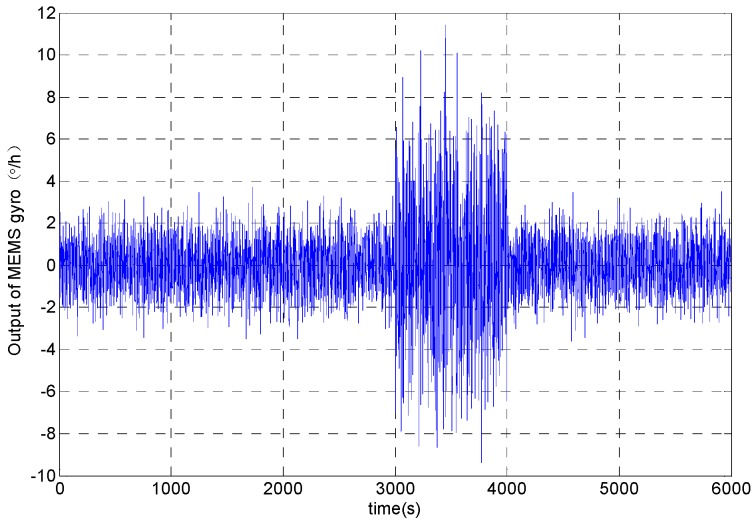
MEMS gyroscope analog signal.

The output signal of MEMS gyroscope is simulated by setting different amplitude Gaussian white noise at different time. The output signal was set the sampling step to 1 s and the sampling time to 6000 s. In the stationary period, the variance of the analog signal is 1 and the variance of the abrupt signal (3000~4000 s) is 3. The analog signal is obtained as shown in Figure 2.

**Figure 3:**
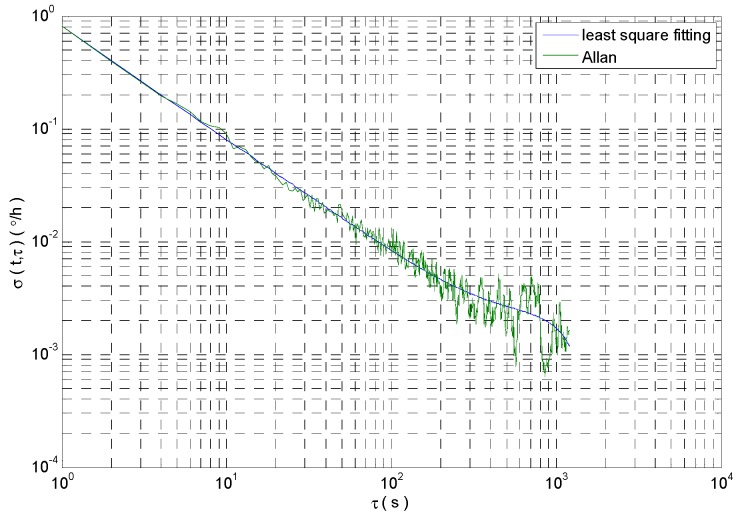
Allan standard deviation curve and least square fitting.

The Allan standard deviation curve is shown in Figure 3, which is obtained by the simulated MEMS gyro signal analysis with Allan variance. A large number of experiments show that different error items usually appear in different time intervals. A certain or a few kinds of random errors play a leading role in different time intervals. Therefore, there is a quantization noise with a slope of −1 in Figure 3. Moreover, angular rate random walk is a kind of random error with long correlation time. Therefore, the random errors such as angular rate random walk and so on are not well represented in Figure 3. Using the least square method to fit the Allan standard deviation curve, the error coefficient of angle random walk can be obtained as 2.36 × 10^−3^°/h^1/2^. The least square fitting results are as shown in Figure 3.

The Allan variance has the advantage of dealing with the ideal stationary signal. But the Allan variance has not strong tracking ability and it has the disadvantage of dealing with the non-stationary signal which is shown in Figure 2. Therefore, many scholars intercept the original signal with window function, and then the intercepted signal was respectively processed with Allan variance analysis. The above method is dynamic Allan variance (DAVAR) [19,20].

### 2.3. Dynamic Allan Variance (DAVAR) Analysis

The basic idea of dynamic Allan variance is firstly to design a proper window function to intercept the original signal. Next, the Allan variance of intercepted signal is calculated separately, and then all the results are combined into a three-dimensional graph to represent the dynamic performance of the signal [19,20]. The principle is as follows:

(1) *t*_1_ is defined as a fixed analysis time *t*, *t* = *t*_1_.
(2) *x*_1_(*t*) is obtained by capturing the original signal with a rectangular window whose width is *T* and center is on *t*_1_*.*
(3) The Allan standard deviation $\sigma (t_{1},\tau )$ is obtained by calculating the clipped signal *x*_1_(*t*).
(4) *t*_2_ is defined as another analysis time point *t*, *t* = *t*_2_. The two interception signals overlap through repeat step (2), and then steps (3) and (4) are repeated, respectively. The Allan standard deviation sequence $\sigma (t_{1},\tau ),\sigma (t_{2},\tau )\cdots \sigma (t_{n},\tau )$ is finally obtained.

In the window section $\left(t-T/2)\leq t^{\prime }\leq (t+T/2\right)$, the original signal is captured using the rectangular window function $P_{\tau }(t)$, and the result is obtained as follows.

(8) $$\omega^{\prime }(t,t^{\prime })=\omega (t^{\prime })P_{\tau }(t-t^{\prime })\;$$

(9) $$P_{\tau }(t)=\{\begin{array}{ll} 1 & \left|t\right|\leq T/2\\ 0 & \mathrm{others} \end{array}\;$$

In the above formula, *t* is the center of the rectangular window and the Allan window $h_{\tau }(t^{\prime })$ is defined as follows.

(10) $$h_{\tau }(t^{\prime })=\{\begin{array}{ll} -1/\tau & 0\leq t^{\prime }<\tau \\ 1/\tau & -\tau \leq t^{\prime }<0 \end{array}\;$$

Increment process $\Delta (t,t^{\prime },\tau )$ is established as in Equation (11).

(11) $$\Delta (t,t^{\prime },\tau )=h_{\tau }(t^{\prime })*\omega_{T}(t,t^{\prime })\;$$

Taking Equation (11) into Equation (1), we get:

(12) $$\sigma^{2}(t,\tau )=\frac{1}{2}<\Delta^{2}(t,t^{\prime },\tau )>\;$$

In summary, the expected value of (12) is defined as the dynamic Allan variance.

(13) $$\sigma^{2}{}_{da\mathrm{var}}(t,\tau )=E(\sigma^{2}(t,\tau ))\;$$

**Figure 4:**
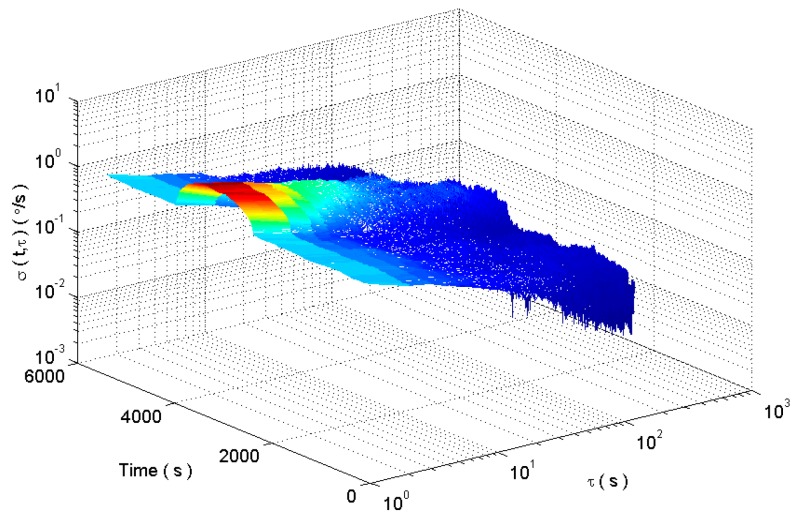
The dynamic Allan variance of analog gyroscope signals.

The square root of (13) is defined as the dynamic Allan standard deviation. Select the window function length of 900. The dynamic Allan variance for the analog signal is shown in Figure 4.

**Figure 5:**
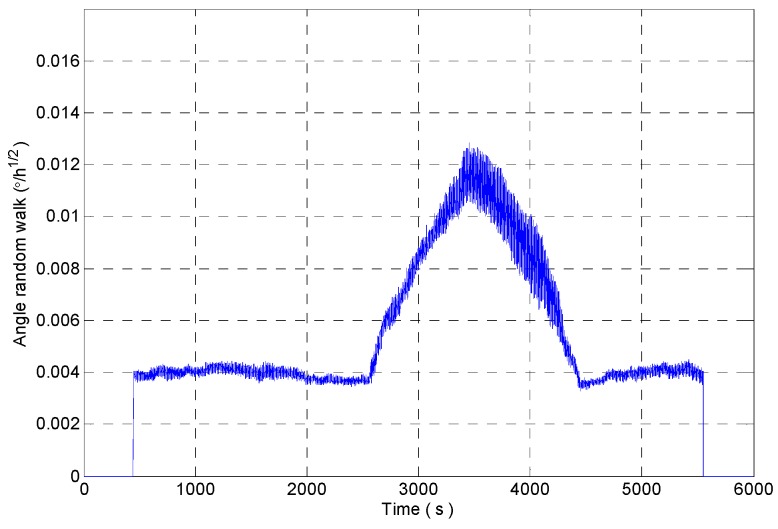
The analysis results of angle random walk.

Figure 4 shows the dynamic Allan variance of the simulated gyroscope signal. In Figure 4, the dynamic Allan variance can show the dynamic change of the signal fluctuation. Although the dynamic Allan variance can track the dynamic performance of the signal, but the tracking result is not perfect from the analysis of the error coefficient in Figure 5. This is because the dynamic Allan variance intercepts the original signal with a fixed-window rectangular window function. There are some shortcomings in the method of intercepting a signal with a fixed window length rectangular window function. If the window length is short, the amount of data per section is small and the confidence of the analysis results is low. If the length of the window is longer, the dynamic changes of the signal are sensed very early and at the same time the dynamic changes disappear very late. As a result, the analysis has the disadvantages of early sensitivity to the beginning and sensitive delay to the end of dynamic signal.

## 3. Kurtosis and Sliding Kurtosis Contribution Coefficient

The kurtosis is a normalized fourth-order central moment, dimensionless and can reflect the distribution of the signal. For a signal of length *n*, the kurtosis coefficient is as in Equation (14).

(14) $$K=\frac{\sum_{i=1}^{n}\left(x_{i}-\mu \right)}{n\sigma^{4}}\;$$

**Figure 6:**
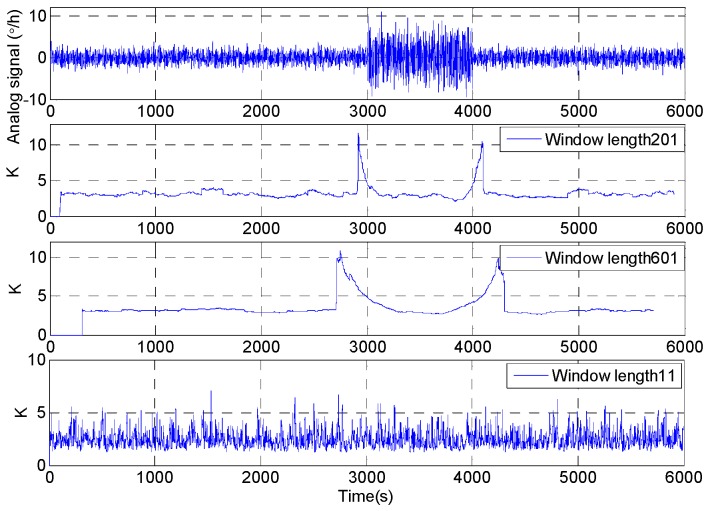
The comparison result of the kurtosis between the different window length.

In Equation (14), *μ* is the mean value of signal $x_{i}$, and σ is the standard deviation of signal $x_{i}$. *K* = 3 (The signal distribution curve has a normal kurtosis); *K* > 3 (The peak value of the impact component in the signal is above the normal distribution curve); *K* < 3 (The impact component amplitude is less than the normal distribution). Wang et al. use rectangular windows to intercept the original signal and then the kurtosis value is calculated to adjust the window length [10,11]. However, the kurtosis of the intercepted signal has the characteristics of a rectangular window intercepting signal, which leads to the shortcomings of early sensitivity to the beginning and sensitive delay to the end of dynamic signal. When using a rectangular window with a window length of 201 and 601, the original signal is intercepted and its kurtosis value is calculated. The kurtosis and the comparison between original signal and the intercepted signal are shown in Figure 6.

Through the above analysis, it is difficult to track the signal fluctuations by using the window function intercepting the signal and then calculating the interception signal kurtosis value. The kurtosis value is more stable with the longer window length, but the worse the tracking results are. In order to accurately track the dynamic characteristics of the signal, the shorter window length of the rectangular window will be selected. When the window length is very short, the kurtosis value is volatile, and it is difficult to track fluctuations of signal. As shown in Figure 6, the kurtosis of window length 11 changes dramatically which make the kurtosis difficult track the dynamic response of the signal. Therefore, this paper proposes the sliding kurtosis contribution coefficient based on the idea of kurtosis contribution coefficient [21]. The principle of sliding kurtosis contribution coefficient is as follows. *K* is kurtosis of the sampling sequence $x_{i}$ (i=1,2,⋯n). *x_ωp_* is obtained by intercepting the original signal with a *L* length rectangular window at the *p* data point in time *t_p_*.

$$x_{\omega p}=\left\{x_{p-\frac{L}{2}},x_{p-\frac{L}{2}+1},\cdots x_{p},x_{p+1},\cdots ,x_{p+\frac{L}{2}-1},x_{p+\frac{L}{2}}\right\}\;$$

In the equation above, *x_mp_* is defined as the sequence in the original signal sequence excluding the *x_ωp_* sequence. Though calculation, the kurtosis of the *x_mp_* sequence is as follows.

$$K_{mp}=\frac{\sum_{i=1}^{n}\left(x_{i}-\mu_{mp}\right)}{(n-L)\sigma_{mp}{}^{4}}(i\neq p-\frac{L}{2},\cdots p,\cdots p+\frac{L}{2})\;$$

In the equation above, *μ_mp_* is the mean value of *x_mp_* and *σ_mp_* is the standard deviation of *x_mp_*. The sliding kurtosis contribution coefficient of sequence *x_ωp_* is as follows.

(15) $$S=\left|K-K_{mp}\right|/K\;$$

**Figure 7:**
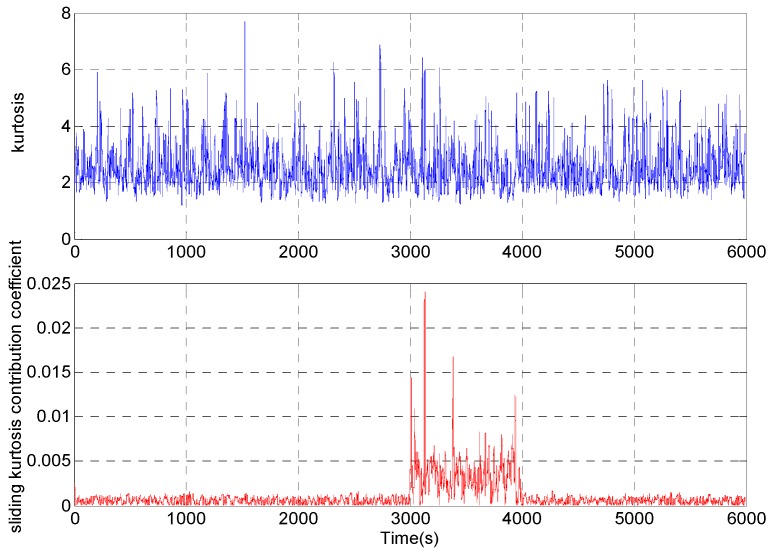
Analysis results of kurtosis and sliding kurtosis contribution coefficient under the same window length.

Sliding kurtosis contribution coefficient of different sequences in different analysis points is calculated, which can accurately estimate the abrupt time of the signal. For the above analog gyro signal, when the length of the rectangular window is *T* = 11, the sliding kurtosis contribution coefficient is shown in Figure 7.

It can be seen from Figure 7 that the sliding kurtosis contribution coefficient which can be applied to the rectangular window with a short length can accurately reflect the dynamic change of the signal.

## 4. Improved DAVAR Algorithm

### 4.1. Extension

When the performance of signal is analyzed with a rectangular window, the problem of low utilization of the data in the beginning and end of the signal is inevitable. In summary, the beginning and end of the signal data is extended with inverted mirror method of extension, which can enhance the utilization of signal interval data.

$$\{\begin{array}{cc} x_{i}^{*}=x_{1-i} & i=-(\frac{L}{2}-1),-(\frac{L}{2}-2),\cdots 0\\ x_{i}^{*}=x_{i} & i=1,2,\cdots N\\ x_{i}^{*}=x_{2N+1-i} & i=N+1,N+2,\cdots N+\frac{L}{2} \end{array}\;$$

When the center of the rectangular window is at $\left[1,\frac{L}{2})\cup (N-\frac{L}{2},N\right]$, the standard deviation of the intercepted sequence is calculated as follows.

$$\sigma (m,L,\tau_{0})=\frac{1}{2{\left(m\tau_{0}\right)}^{2}(L-2)}\times \sum_{n=2}^{\frac{L}{2}-1}{\left(x_{n-m}^{*}-2x_{n}^{*}+x_{n+m}^{*}\right)}^{2}\;$$

$$\sigma (m,L,\tau_{0})=\frac{1}{2{\left(m\tau_{0}\right)}^{2}(L-2)}\times \sum_{n=N-\frac{L}{2}}^{N}{\left(x_{n-m}^{*}-2x_{n}^{*}+x_{n+m}^{*}\right)}^{2}\;$$

In the equation above, *L* is the window length of the rectangular window, *m* generally take 1/3 the length of the data column, $\tau_{0}$ is the sampling time.

### 4.2. Improved Window Length Adjusting Adaptively Algorithm

The traditional DAVAR algorithm intercept the original signal using a fixed window length rectangular window and analyze the dynamic Allan variance of the intercepted data, which has disadvantages of early sensitivity to the beginning and sensitive delay to the end of dynamic signal. A rectangular window function is designed by combining slipping kurtosis contribution coefficient and kurtosis. The rectangular windows with longer window lengths are used in the smoothing interval of the signal, which can enhance the confidence and stability of the analysis results. If the signal in the strong changes interval, the signal is dealt with short-window length rectangular window, which can accurately track the dynamic changes of signals and improve the tracking ability of dynamic Allan variance. So the improved window length adjusting adaptively algorithm is as follows.

(1) The *L* window is defined as a rectangular window with a suitable window length of *L*. Using the *L* window to intercept the original signal and analyze the kurtosis of the intercepted signal. The kurtosis threshold for stable signal is [*K*_1_,*K*_2_], such that at the analysis moment *t_p_*, the kurtosis value of this sequence *K_p_* is calculated and the following relation is obtained.

(16) $$f_{1}(t_{p})=\{\begin{array}{cc} 0 & K_{p}\in [K_{1},K_{2}]\\ 1 & K_{p}\notin [K_{1},K_{2}] \end{array}\;$$

(2) The *l* window is defined as a rectangular window with a suitable window length of *l* (*l* < *L*). The slipping kurtosis contribution coefficient of the signal sequence that is obtained by intercepting the signal from the end of a sequence cut from *L* window with *l* window is calculated. Through calculating the kurtosis value *K_mp_* of the front *L* − *l* length signal in the *L* window intercepting signal, the sliding kurtosis contribution coefficient $S=\left|K_{p}-K_{mp}\right|/K_{p}$ of the *l* window is calculated. *S* reflects the stability of the signal at the end of the *L* window and achieves an accurate sensitivity to the end of the intercepted signal sequence. The sliding kurtosis contribution coefficient in [*S*_1_,*S*_2_] is designed to represent the stable signal, which leads to the existence of Equation (17) on the *t_p_* moment.

(17) $$f_{2}(t_{p})=\{\begin{array}{cc} 0 & S\in [S_{1},S_{2}]\\ 1 & S\notin [S_{1},S_{2}] \end{array}\;$$

The function *f*_2_ reflects whether there is an impact component at the $t_{p}+\frac{L-l}{2}\tau_{0}$ moment. Where $\tau_{0}$ is the sampling interval.

(3) The above two output function values are combined and then get the following relationship.

(18) $$f(t_{p})=f_{1}(t_{p})f_{2}(t_{p})\;$$

When *f*(*t_p_*) is 0, the characterization of the signal is stationary, and the end of the intercept signal contains no impact components. When *f*(*t_p_*) is 1, the signal is unstable, and the rising edge of *f*(*t_p_*) is collected, which is known as the impact component, exists at time $t_{p}+\frac{L-l}{2}\tau_{0}$.

(4) When the dynamic Allan analysis of variance is analyzed, the maximum window length *L*_max_ for a steady signal and the minimum window length *L*_min_ for an unstable signal are usually designed. When *f*(*t_p_*) is 1, the signal contains the impact component at the $t_{p}+\frac{L-l}{2}\tau_{0}$ moment, and the *L* window starts to capture the signal at time $t_{p}+\frac{L-l}{2}\tau_{0}$ with the minimum window length *L*_min_. Assuming that the initial window length of the *L* window is $L^{\prime }$, the maximum distance of the center about the *L* window moving distance is $\frac{L^{\prime }-L_{\min}}{2}$, and the design of the window length variation factor *∆L*_1_ is as follows.

(19) $$\Delta L_{1}\geq \frac{L^{\prime }-L_{\min}}{\frac{L^{\prime }-L_{\min}}{2}}=2\;$$

When *f*(*t_p_*) = 1, and at the next point *t_p_*_+1_, the *L* window’s window length *L*(*t_p_*_+1_) is as follows:

(20) $$L(t_{p+1})=\{\begin{array}{cc} L_{\min} & L(t_{p})\leq L_{\min}\\ L(t_{p})-\Delta L_{1} & L_{\min}<L(t_{p})<L_{\max}\\ L_{\max} & L(t_{p})\geq L_{\max} \end{array}\;$$

When $f(t_{p})=0$, the intercepted signal is stable and the starting point $t_{p}-\frac{L^{\prime }}{2}\tau_{0}$ of the intercepted sequence contains no impact component. In this case, it is required that L window after increasing the window length can not acquire the signal before the $t_{p}-\frac{L^{\prime }}{2}\tau_{0}$ time again. At this time, the window length of the *L* window is increased until it reaches *L*_max_. The maximum distance of *L* window center point moving is $\frac{L_{\max}-L^{\prime }}{2}$, then the window length variation factor *∆L*_0_ is designed at this time.

(21) $$\Delta L_{0}\geq \frac{L_{\max}-L^{\prime }}{\frac{L_{\max}-L^{\prime }}{2}}=2\;$$

And at the next point *t_p_*_+1_, the *L* window’s window length *L*(*t_p_*_+1_) is as follows.

(22) $$L(t_{p+1})=\{\begin{array}{cc} L_{\min} & L(t_{p})\leq L_{\min}\\ L(t_{p})+\Delta L_{0} & L_{\min}<L(t_{p})<L_{\max}\\ L_{\max} & L(t_{p})\geq L_{\max} \end{array}\;$$

## 5. Simulation and Test

**Figure 8:**
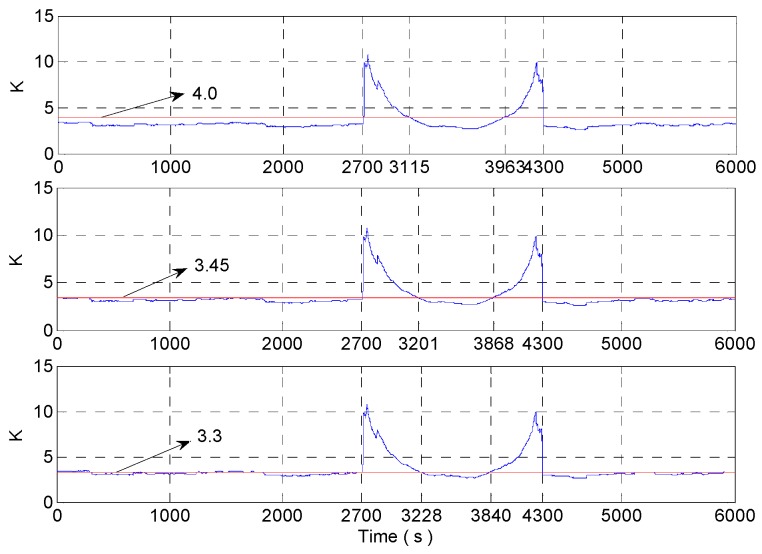
The analysis of the different stability thresholds of kurtosis.

Taking the rectangular window with a window length of 600 as an example, the adaptive change of window length based on kurtosis is analyzed as follows. The analysis results are as in Figure 8. If the stability threshold of kurtosis is 4, and the sensitive nodes of dynamic signals are 2700 s, 3115 s, 3963 s, and 4300 s. When kurtosis is used to control the change of window length, reduce the length of the window at 2700 s, and the beginning of the rectangle window just collects the 3000 s data; increase the length of window at 3115 s, the end of the rectangular window collects the 2815 s data (3000 s data should be collected), and the signal is in a stable range, so the adjustment of window length is obviously too advanced. When the rectangle window is at 3963 s, the window length begins to decrease to sense the dynamic change of 4000 s data. But the 4263 s data is collected at the beginning of the rectangular window, and the signal is already in a stable state at this time, so there is a serious lag phenomenon in adaptive adjustment of window length.

If the stability threshold of kurtosis is 3.45, the sensitive nodes of dynamic signals are 2700 s, 3201 s, 3868 s, 4300 s. When kurtosis is used to control the change of window length, reduce the length of the window at 2700 s, and the beginning of the rectangle window just collects the 3000 s data; increase the length of window at 3201 s, the end of the rectangular window collects the 2901 s data (3000 s data should be collected), and there is still an adjustment ahead of time. When the rectangle window is at 3868 s, the window length begins to decrease to sense the dynamic change of 4000 s data. But the 4168 s data is collected at the beginning of the rectangular window, and there is also a lagging control.

If the stability threshold of kurtosis is 3.3, the rectangle window begins to increase the length of the window at 3228 s, and the end of the rectangular window collects the 2928 s data (3000 s data should be collected); the rectangle window begins to reduce the length of the window at 3840 s, and the 4140 s data is collected at the beginning of the rectangular window.

So the dynamic tracking effect is best when the stability threshold of kurtosis is 3.3. But at this time, the kurtosis value of the stationary interval has exceeded the kurtosis stability threshold, that is, the threshold value of the kurtosis is relatively small. From Figure 8, we can see that the closer the kurtosis threshold is to 3, the more sensitive the adjustment of the rectangular window is, and the better the dynamic tracking effect is. Because in 3000~4000 s, the variance of the noise is increased to 3, that is, there is a sudden increase in the amplitude of noise at 3000 s. According to Equation (14), we know that the kurtosis value at 3000 s (the center of the rectangular window is in 2700 s) is positively correlated with the difference of noise variance before and after mutation. When the rectangular window intercepts the abrupt signal, the kurtosis will decrease gradually, no matter how large the variance of the mutation signal is. So the kurtosis value changes slowly between 3000~4000 s. The slow change leads that the adjustment of rectangular windows based on kurtosis is insensitive in this area. The sliding kurtosis contribution coefficient is used to characterize the signal in the kurtosis slowly changing region. So the stability threshold of kurtosis can be achieved closer to 3, and the better the dynamic tracking ability of improved DAVAR will be.

**Figure 9:**
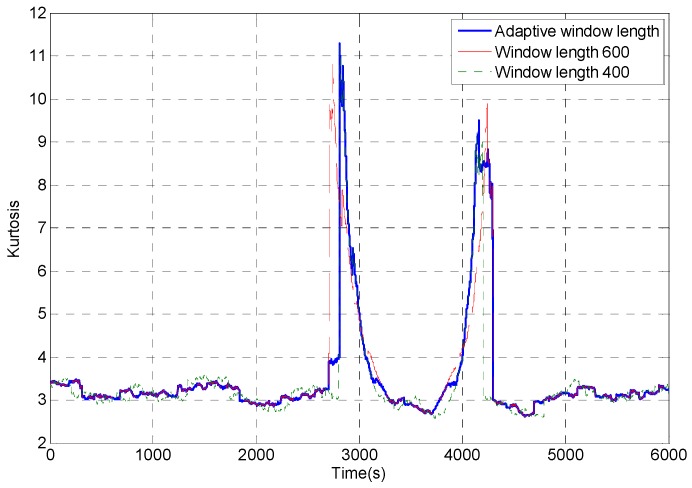
Kurtosis value of the intercepted signal with different window length.

According to the analog MEMS gyroscope designed in Section 2.2, the random error of signal is analyzed by the improved dynamic Allan variance method. Setting parameters: *L*_max_ = 600, *L*_min_ = 400; *K*_1_ = 2.5, *K*_2_ = 3.3; *S*_1_ = 0, *S*_2_ = 0.07; *l* = 100; *∆L*_0_ = *∆L*_1_ = 2. The *l* window’s window length and above parameters can be adjusted according to the sensitivity of the impact components in signal. The kurtosis value of the captured signal which is obtained by capturing original signal with rectangular windows with window length of 600, 400, or adaptive window length are obtained, respectively, as shown in Figure 9. It can be found that before the impact signal is intercepted, the kurtosis value of the adaptive window length rectangular window intercepting signal coincides with the kurtosis value of the 600 window length rectangular window intercepting signal. And after the impact signal, the kurtosis value of the adaptive window length rectangular window intercepting signal coincides with the kurtosis value of the 400 window length rectangular window intercepting signal.

**Figure 10:**
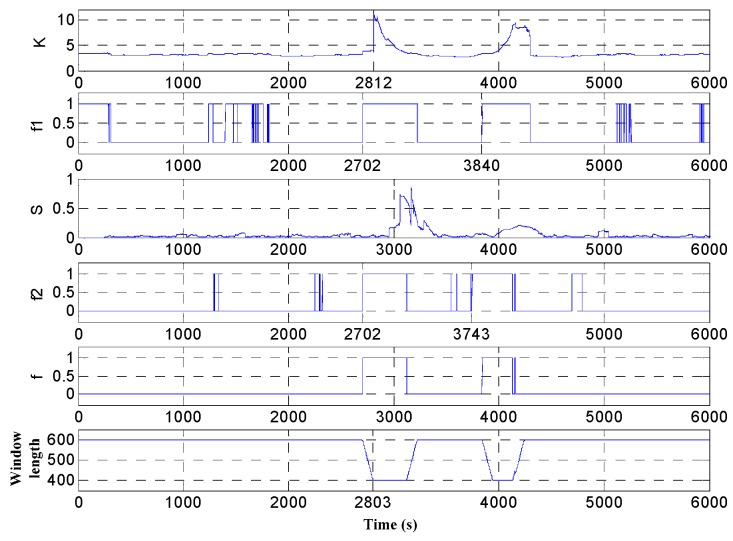
Simulation result.

The simulation test is carried out according to the above parameters. And the signal kurtosis, *f*_1_, sliding kurtosis contribution coefficient, *f*_2_, *f* and adaptive window length is shown in Figure 10. It can be seen from Figure 10 that the adaptive window length becomes the minimum window length at the 2803 s, and the data of the 3003 s is intercepted which is at the end of the intercepted signal and which is the starting point of the shock signal. Compared with the sliding kurtosis contribution coefficient, kurtosis can characterize the signal stability better, but it is insensitive to the end position of the impact component in the signal. As shown in Figure 10, the interval [*K*_1_,*K*_2_] is relatively small (Some slightly fluctuating signals are shown unstable and *f*_1_ jumps to 1). In this case, the shock signal is only perceived when the *L* window reaches 3840 s. And at this time, the end of the *L* window intercepting signal is the data at 4140 s. So the design of adaptive window length simply according to kurtosis is difficult to meet the requirements. Compared to the sensitivity of the sliding kurtosis contribution coefficient, the *L* window is already sensitive to the end of the shock signal when it reaches 3743 s. And at this time, the end of the *L* window intercepting signal is the data at 4043 s. Therefore, the reduction of window length at this time can improve the sensitivity of the impact component.

**Figure 11:**
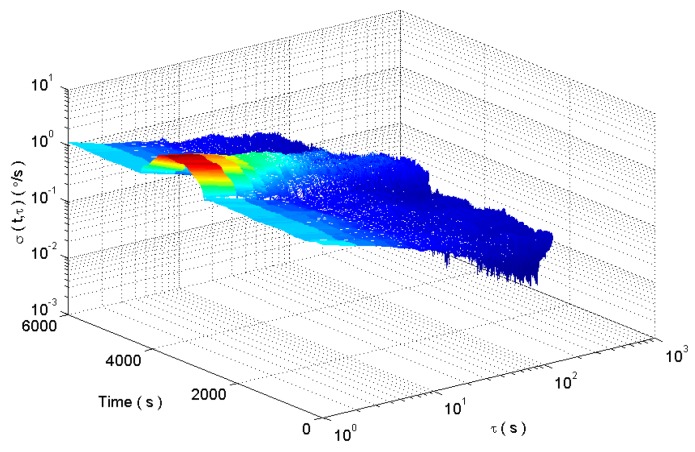
The analysis results of analog signals based on improved DAVAR algorithm.

According to the improved DAVAR method, the dynamic Allan variance of the analog signal is shown in Figure 11.

**Figure 12:**
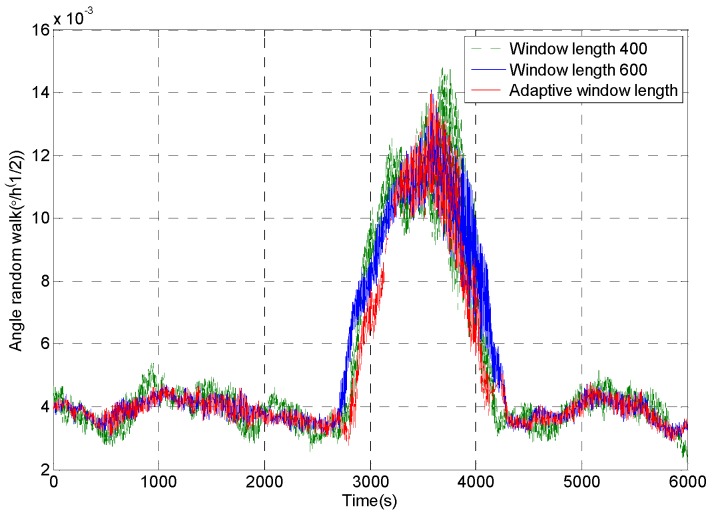
The angle random walk analysis results of intercepted signal of a rectangular window with different window lengths.

Figure 12 shows the random walk analysis results of the signal sequences which are obtained through intercepting the original signal by using the rectangular windows with different window lengths. It can be seen that the rectangular window with the window length of 600 is sensitive to the impact signal very early, and the recovery is later. The rectangular window with the window length of 400 is more sensitive to the impact signal, but the analysis result fluctuates obviously. The rectangular window with adaptive window length can accurately detect the impact component in the signal, and it ensures the stability of the analysis results.

The angle random walk of gyro is identified by improved DAVAR method. The comparison of confidence level and dynamic tracking ability of the analysis results between improved DAVAR (Adaptive window length DAVAR, A-DAVAR), DAVAR-400 (window length is 400) and DAVAR-600 (window length is 600) is shown in Table 1.

In Table 1, the beginning (3000 s) means that the window first senses the 3000 s data, and the window length should decrease at this time. The beginning (3000 s) should be close to DAVAR-600’s beginning (3000 s). The end (3000 s) means that the window lost senses the 3000 s data, and the window length should increase at this time. The end (3000 s) should be close to DAVAR-400’s end (3000 s). The ARW (0~2000 s) means the mean value from 0 s to 2000 s and the region is in stationary interval, so the ARW (0~2000 s) should be close to the refer value in Section 2.2.

From Table 1, we can see that the improved dynamic Allan variance solves the problem of both confidence and dynamic tracking ability.

The MEMS gyro is sensitive to temperature, so the MEMS gyro is fixed to the stationary preheating 1 h on the turntable to make the temperature reach steady state. The accurate angular velocity can be input to the MEMS gyro using the turntable. But the input angular velocity of the turntable is 0 in the experiment. Since the gyroscope bandwidth is 40 Hz, the sampling frequency is 100 Hz according to sampling theorem.

**Figure 13:**
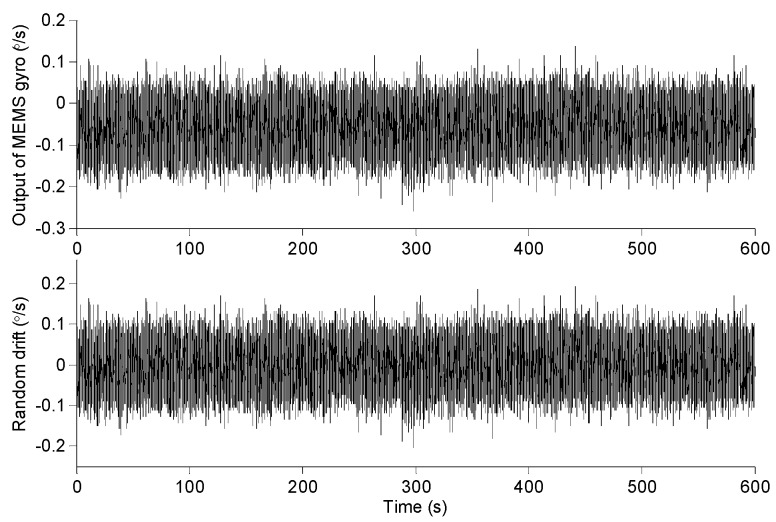
The original output and random drift of MEMS gyroscope.

The gyro data collected for the 600 s is shown in Figure 13. When the turntable input angle speed is 0, the output signal of MEMS gyroscope contains trend and random drift. Usually the mean value method and polynomial fitting method are used to remove the trend terms. So the random drift of MEMS gyro is obtained by removing trend items from data collection with 3-order polynomial fitting method. The random drift is shown in Figure 13.

**Figure 14:**
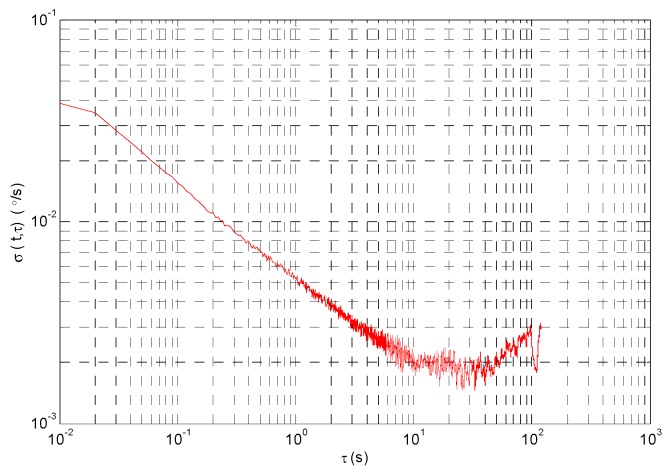
Allan standard deviation curve.

The random drift of gyroscope is analyzed with Allan variance, and the Allan standard deviation curve is shown in Figure 14. The Figure 14 shows that the random error includes quantization error, zero bias instability, angular random walk and angular rate random walk. Fitting the Allan standard deviation curve by least square method, the error coefficient of angular random walk is 0.355°/h^1/2^.

**Figure 15:**
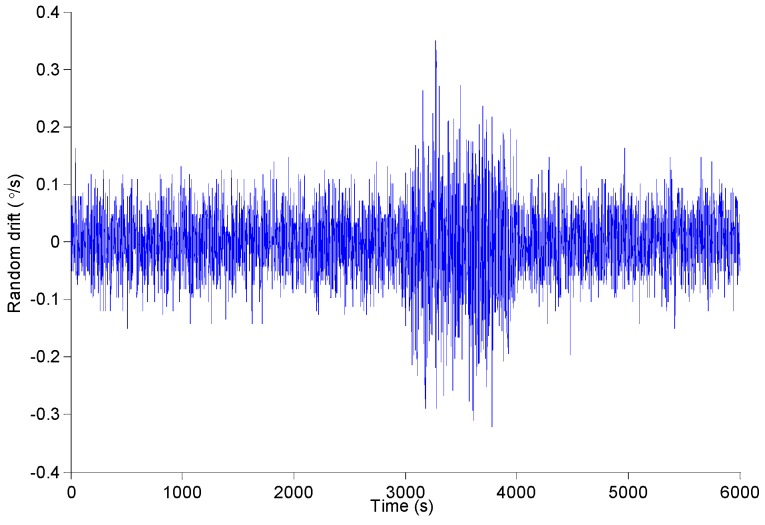
The dynamic signal of MEMS gyroscope.

**Figure 16:**
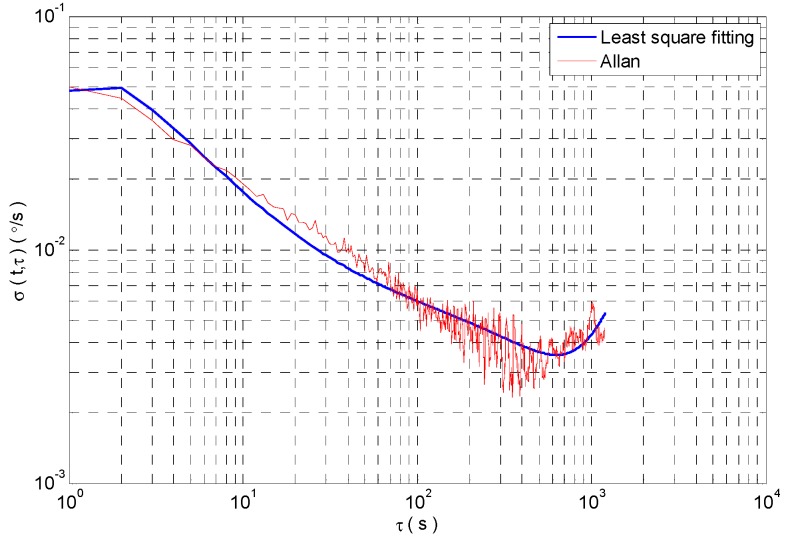
Allan standard deviation curve and least square fitting curve.

In the experiment, a slight disturbance is exerted to the gyroscope at 3000~4000 s. The sampling frequency is 100 Hz. The sampling time is 6000 s. According to a rule, one of 100 consecutive sampling data is selected as the research sample. And the gyro signal is shown in Figure 15. The Allan standard deviation curve and the least square fitting curve of the MEMS gyroscope are shown in Figure 16. We can get the error coefficient of random error by least square fitting. However, the analysis results can not reflect the dynamic changes of signals.

**Figure 17:**
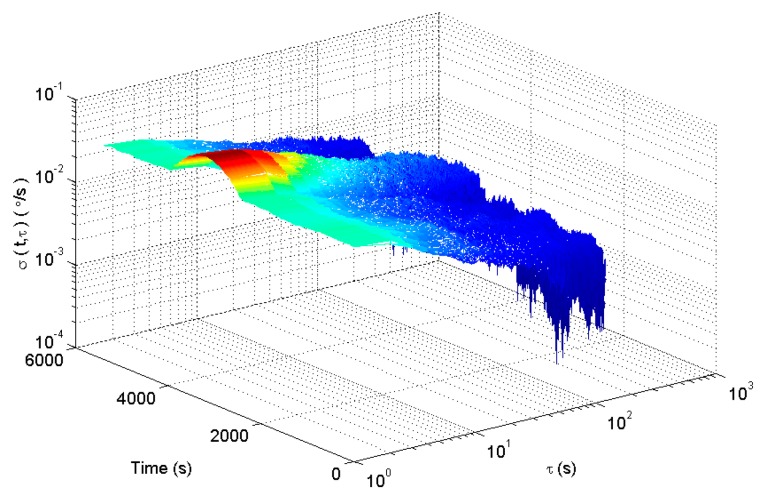
The dynamic Allan variance analysis.

The rectangle window length is chosen as 600. The dynamic Allan variance of MEMS gyro is obtained as shown in Figure 17.

**Figure 18:**
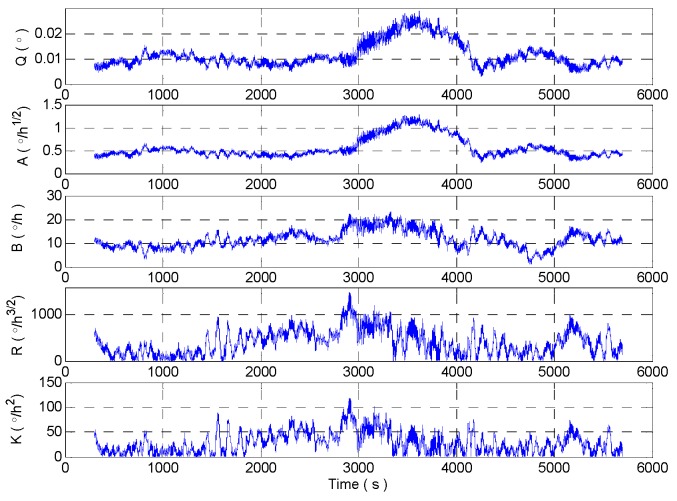
The random error analysis results of MEMS gyroscope.

The quantization noise (QN, Q is a coefficient), angle random walk (ARW, A is a coefficient), bias instability (BI, B is a coefficient), rate random walk (RRW, R is a coefficient), rate ramp (RR, K is a coefficient) are obtained by fitting Allan standard deviation curve with least squares. And the random error is shown in Figure 18. We can see that the random error analysis results show strong dynamic characteristics.

**Figure 19:**
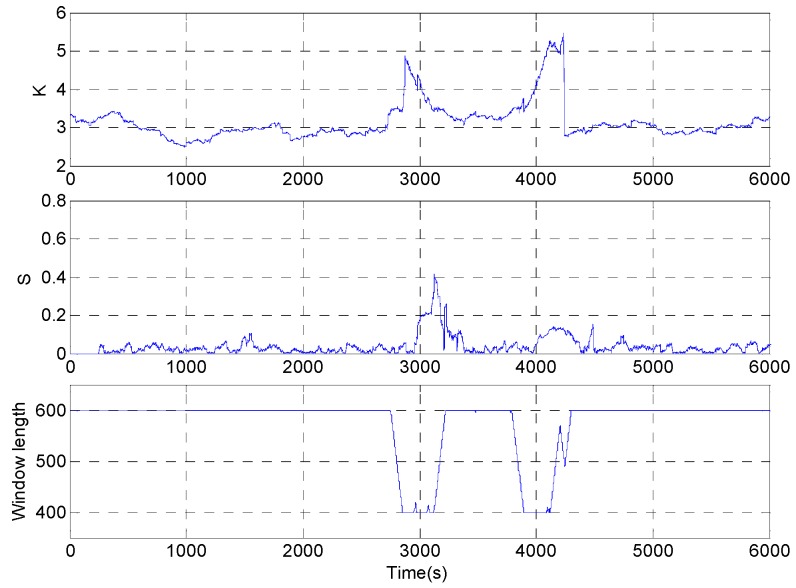
The analysis result of kurtosis, sliding kurtosis contribution coefficient, and adaptive window length.

According to the improved DAVAR algorithm designed above, the sampled signal is analyzed. The kurtosis, sliding kurtosis contribution coefficient, and adaptive window length are shown in Figure 19.

**Figure 20:**
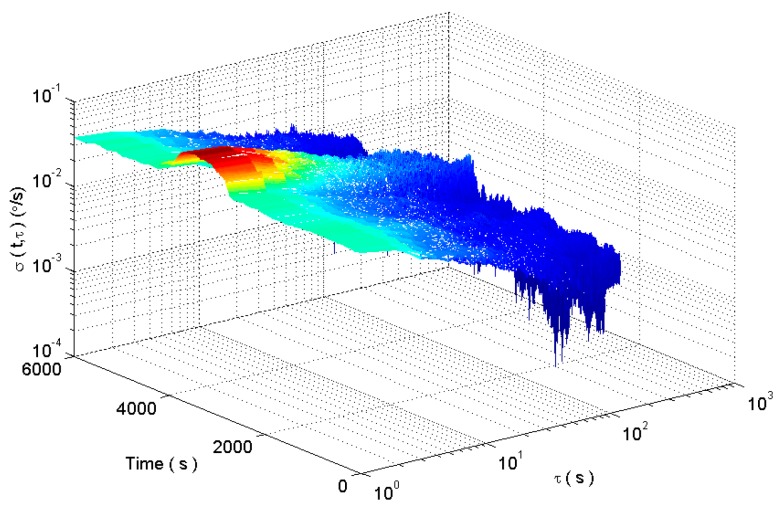
DAVAR analysis results.

Figure 19 shows that when the beginning of shock signal is collected by the *L* window at 3000 s, the length of the rectangular window begins to decrease gradually until the length of the window is reduced to a minimum *L*_min_. And when the beginning of the shock signal is collected by the end of the rectangular window, the length of the rectangular window increases gradually to the maximum length *L*_max_. In the interval of the end of the shock signal, there is a slight fluctuation in the signal for a short time due to temperature and electromagnetic interference which leads to reduce the length of the rectangular window in the improved DAVAR method. The sliding kurtosis contribution coefficient highlights the more sensitive ability, such as the peak in Figure 19. And the window length decreases in the growth interval after 4000 s, so the tracking ability of dynamic Allan variance has improved by the window length decreasing. The measured signals are analyzed with the improved DAVAR method, and the dynamic Allan variance analysis results of the measured signals are as shown in Figure 20.

**Figure 21:**
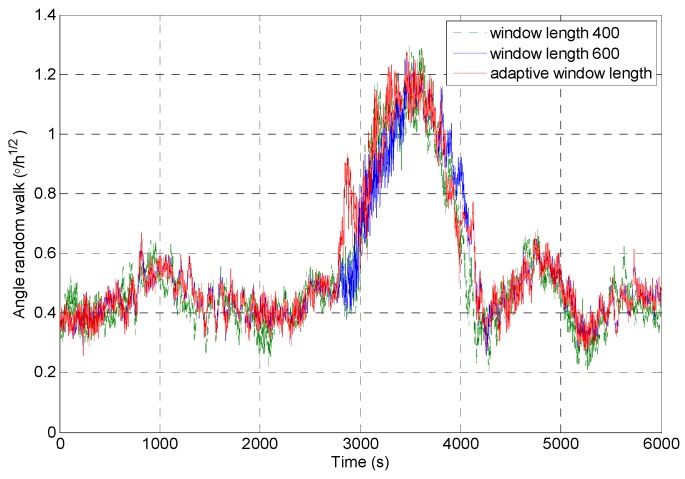
Angle random walk analysis results of rectangular windows with different window length.

The feasibility of the improved DAVAR algorithm is analyzed by taking the angle random walk as the analysis example. The dynamic changes of the angular random walk error coefficient of the measured MEMS gyroscope signal are shown in Figure 21. We can find that the improved DAVAR algorithm with adaptive window length can be very sensitive to impact signal. And in the stationary interval (0~3000 s, 4000~6000 s), the improved DAVAR analysis results coincide with the DAVAR analysis results obtained from the rectangular window interception signal with a window length of 600. The results of the analysis have good confidence and stability. In the perturbation interval (3000~4000 s), the improved DAVAR analysis results coincide with the DAVAR analysis results obtained from the rectangular window interception signal with a window length of 400. The results of the analysis have good confidence and stability. And the analysis results have good dynamic tracking ability. So the feasibility of the proposed method is verified.

The error of gyro angle random walk is identified by improved DAVAR method. The comparison of confidence level and dynamic tracking ability of the analysis results between improved DAVAR (A-DAVAR), DAVAR-400, and DAVAR-600 is as shown in Table 2. The Refer value is obtained by the Allan variance in Figure 16.

**Figure 22:**
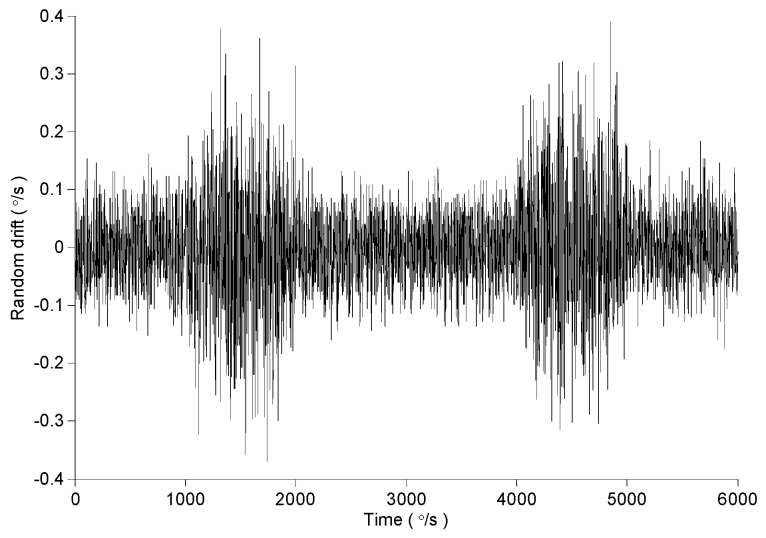
The dynamic random drift of MEMS gyroscope.

In order to further verify the feasibility of the proposed method, change the time of mechanical interference. And the interference is introduced into the gyroscope test at 1000~2000 s and 4000~5000 s. Remove the trend items from the original output and the dynamic random drift is obtained, as shown in Figure 22.

**Figure 23:**
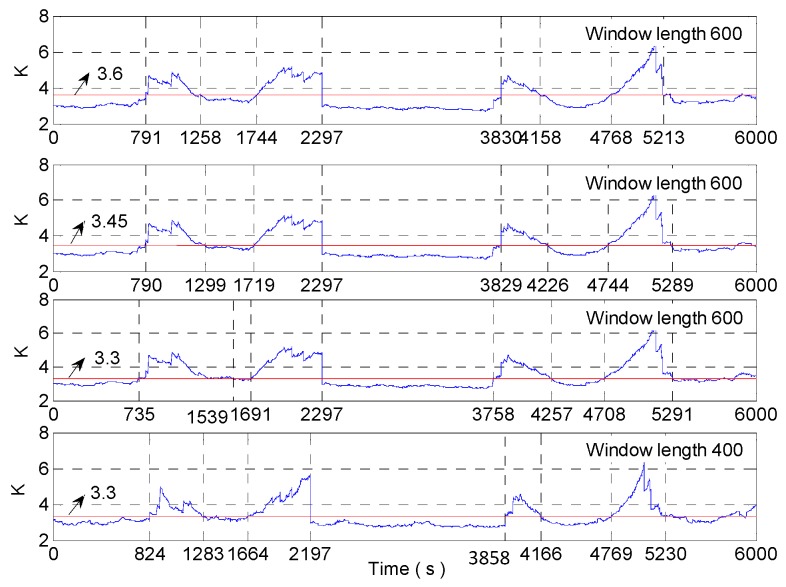
The analysis of the different stability thresholds of kurtosis.

If the kurtosis threshold is 3.6, 3.45 and 3.3, the rectangular window with a window length of 600 and 400 is used to intercept signal for dynamic Allan variance analysis. The nodes obtained by the window length adjustment according to kurtosis are shown in Figure 23. As shown in Figure 23, the closer the kurtosis threshold is to 3, the more sensitive the adjustment will be. But if the kurtosis threshold is too close to 3, the judgement of the stationarity of the signal will be distorted according to kurtosis. For example, when the length of the window is 600 and the kurtosis threshold is 3.3, 735~1539 s is judged to be unstable, and there is a big misjudgement. Therefore, we need to combine the sliding kurtosis contribution coefficient and kurtosis together to determine the stable form of the signal.

**Figure 24:**
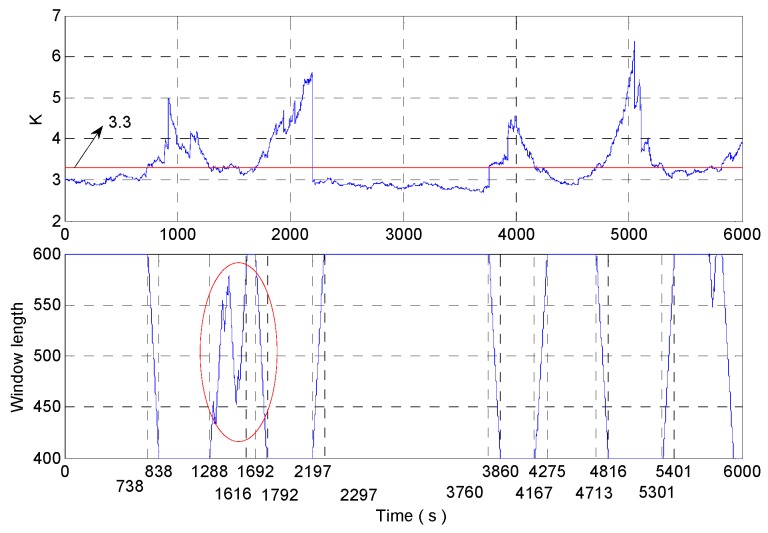
The kurtosis and the window length in DAVAR-K-3.3.

We can see the dynamic tracking ability of analysis results is best when the stability threshold of kurtosis is 3.3 in Figure 23. So adjust the length of a rectangular window only based on kurtosis (brief account DAVAR-K-3.3) as the same as Wang’s method [12,13]. The kurtosis and the window length are shown in Figure 24. In Figure 24, the stability threshold of kurtosis is 3.3 and the window length in the red circle is not perfect. The ideal situation is that the signal should be intercepted by the rectangle window with 600 window length from 0s to 700 s. At 700 s, the beginning of the rectangular window begins to intercept the 1000 s signal, so the length of the window begins to decrease and the beginning of the rectangular window has been at 1000 s. When the rectangle window moves to 800 s, the length of the window is minimized to 400, at this time the beginning of the rectangular window is still in 1000 s. Then use the rectangular window with a length of 400 to intercept the signal after 1000 s until the movement reaches 1200 s. At 1200 s, the end of the rectangular window begins to intercept the 1000 s signal, the rectangle window length begins to increase, and the end of the rectangular window has been at 1000 s until it moves to 1300 s. And the length of the window grows to the maximum at 1300 s, at this time the end of the rectangular window is still located in 1000 s. The signal should be intercepted by the rectangle window with a 600 window length from 1300 s to 1700 s. This is the most ideal case, with the strongest sensitivity and the highest confidence level at every moment. The process of changing the length of the window is the same as that of the process mentioned above. So the window length in the red circle region influences the analysis results because of the small stability threshold of kurtosis.

**Figure 25:**
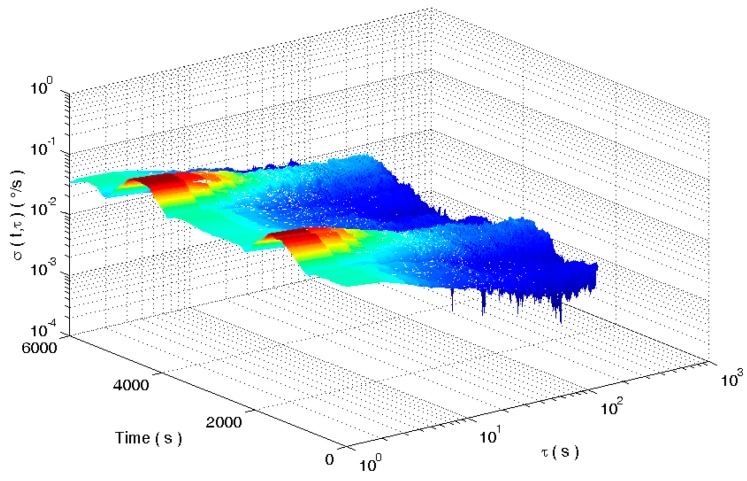
The dynamic Allan variance analysis results in DAVAR-K-3.3.

**Figure 26:**
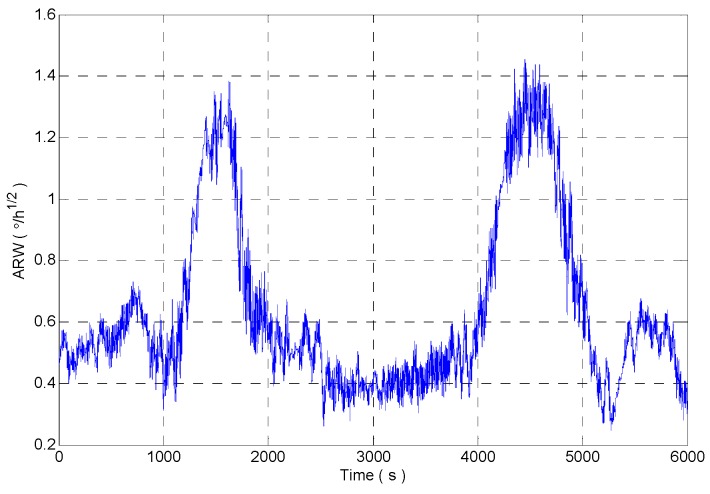
The angle random walk in DAVAR-K-3.3.

The dynamic Allan variance analysis results with DAVAR-K-3.3 are shown in Figure 25. And the angle random walk obtained by the DAVAR-K-3.3 is shown in Figure 26.

**Figure 27:**
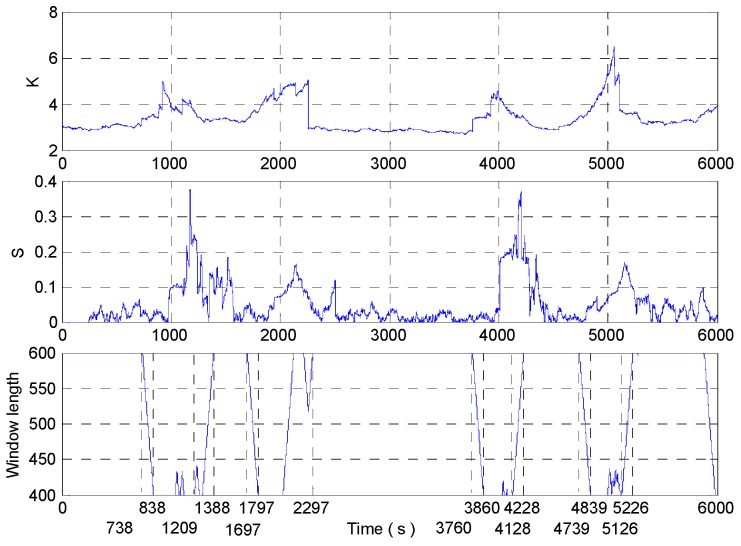
Analysis result.

Do not change the parameters in the algorithm and the sampled signals are analyzed with the improved DAVAR algorithm. The analysis results of kurtosis, sliding kurtosis contribution coefficient and the window length are shown in the Figure 27. In Figure 27, the window length has been achieved adjust adaptively and the window length adjustment is close to the ideal situation.

**Figure 28:**
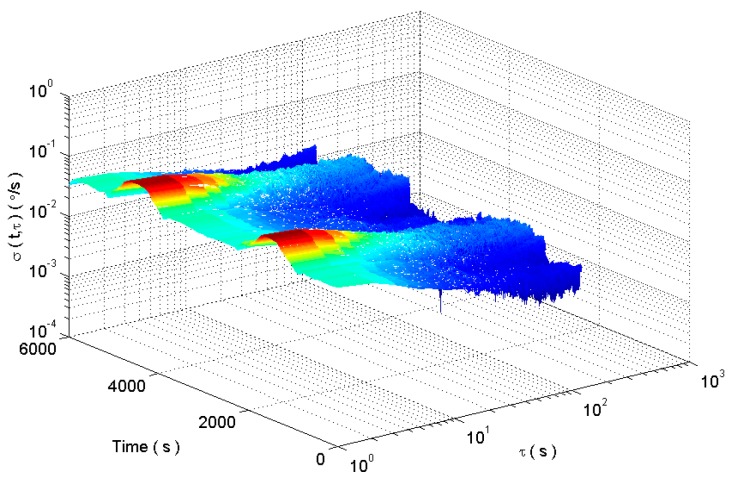
The dynamic Allan variance analysis results.

The improved dynamic Allan variance analysis of dynamic random drift signals is shown in the Figure 28.

**Figure 29:**
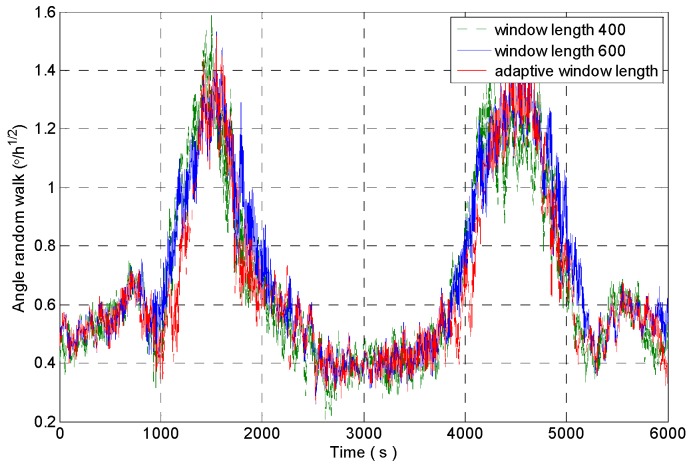
Angle random walk analysis results.

Taking the angle random walk as an example, the validation of the proposed method is carried out, and the analysis result is shown in Figure 29. As shown in Figure 29, the analysis results of the improved DAVAR algorithm have stronger sensitivity than the DAVAR-600, and they are more stable than the results of DAVAR-400.

The comparison of confidence level and dynamic tracking ability of the analysis results between improved DAVAR, DAVAR-400, DAVAR-600, and DAVAR-K-3.3 is shown in Table 3.

In Table 3, the beginning (1000 s) means that the window first sense the 1000 s data, and the window length should decrease at this time. The beginning (1000 s) should be close to DAVAR-600’s beginning (1000 s). The end (1000 s) means that the window lost sense the 1000 s data, and the window length should increase at this time. The end (1000 s) should be close to DAVAR-400’s end (1000 s). The restore 600 (1000 s) means the window length restores to 600 after 1000 s, and the window begins to intercept the signal in stable region after 1000 s, so the restore 600 (1000 s) should be close to the DAVAR-600’s end (1000 s). Other nodes are similar to the above. ARW (2500~3500 s) means the mean value of ARW from 2500 s to 3500 s and, in the stationary interval, the ARW should be close to the refer value.

From Table 3 and the analysis above, we can see that the improved dynamic Allan variance solves the problem of both confidence and dynamic tracking ability and the analysis results are the closest to the ideal situation in A-DAVAR, DAVAR-400, DAVAR-600, and DAVAR-K-3.3.

## 6. Conclusions

This article elaborates the shortcomings of Allan variance and dynamic Allan variance by using these two methods to analyze the analog MEMS gyro signal. Because the dynamic Allan variance analysis using the rectangular window with a fixed window length to capture the original signal has the disadvantage of power leakage and insensitive tracking of the impact signal. In this paper, the DAVAR analysis of signal with a rectangular window with fixed window length is used to analyze the signal. When the window length is long, the dynamic tracking ability of the signal is poor, but the reliability is high. On the contrary, when the window length is small, the dynamic tracking ability is strong but the confidence level is poor. Using kurtosis to adjust the length of the rectangle window, the method can improve the dynamic tracking ability of the analysis results. But by comparing and analyzing the different kurtosis stability threshold, it is found that the more closer the kurtosis stability threshold is close to 3, the stronger the dynamic tracking ability of DAVAR will be. However, when the kurtosis stability threshold is too close to 3, there is a misjudgement in kurtosis analysis of signal stability, resulting in distortion of DAVAR analysis results. So the slipping kurtosis contribution coefficient is proposed to deal with the impact component at the end of sensitive signals. And the paper carry out the beginning and end interval data of the signal with an inverted mirror extension to increase the utilization of beginning and end data. Through the kurtosis and sliding kurtosis contribution coefficient jointly adjusting the window length of the rectangular window, the kurtosis stability threshold can be closer to 3, which improves the dynamic tracking ability of the analysis results and realizes the adaptive change of the length of the window. It solves the problem that the dynamic Allan variance tracking ability and confidence level are difficult to take into account, and also solves the problem of misjudgement in the stability analysis of kurtosis.

## Figures and Tables

**Table 1:** The comparison of confidence level and dynamic tracking ability of the analysis results between A-DAVAR, DAVAR-400, and DAVAR-600.

Analysis Node	DAVAR-400	DAVAR-600	A-DAVAR
ARW (10^−3^°/h^1/2^) (0~2000 s)	4.03	3.87	3.83
ARW (10^−3^°/h^1/2^) (3000~4000 s)	10.97	10.32	9.45
Beginning (3000 s)	2800	2702	2703
End (3000 s)	3200	3300	3156
Beginning (4000 s)	3800	3700	3740
End (4000 s)	4200	4300	4200

**Table 2:** The comparison of confidence level and dynamic tracking ability of the analysis results between A-DAVAR, DAVAR-400 and DAVAR-600.

Analysis Node	DAVAR-400	DAVAR-600	A-DAVAR
Refer value (°/h^1/2^)	0.355	0.355	0.355
ARW (°/h^1/2^) (0~2000 s)	0.518	0.476	0.451
ARW (°/h^1/2^) (3000~4000 s)	1.083	1.032	1.025
Beginning (3000 s)	2800	2702	2713
End (3000 s)	3200	3300	3189
Beginning (4000 s)	3800	3700	3723
End (4000 s)	4200	4300	4176

**Table 3:** The comparison of confidence level and dynamic tracking ability of the analysis results between A-DAVAR, DAVAR-400, and DAVAR-600.

Analysis Node	DAVAR-400	DAVAR-600	DAVAR-K-3.3	A-DAVAR
Refer value (°/h^1/2^)	0.355	0.355	0.355	0.355
ARW (°/h^1/2^) (1000~2000 s)	1.136	1.091	1.107	0.963
ARW (°/h^1/2^) (2500~3500 s)	0.423	0.385	0.389	0.383
ARW (°/h^1/2^) (4000~5000 s)	1.172	1.105	1.086	1.061
Beginning (1000 s)	800	700	738	738
End (1000 s)	1200	1300	1288	1209
Restore 600 (1000 s)	-	-	1616	1388
Beginning (2000 s)	1800	1700	1616	1697
End (2000 s)	2200	2300	2297	2215
Beginning (4000 s)	3800	3700	3760	3760
End (4000 s)	4200	4300	4167	4128
Beginning (5000 s)	4800	4700	4713	4739
End (5000 s)	5200	5300	5301	5126
